# Engineering Electrode Rinse Solution Fluidics for Carbon-Based Reverse Electrodialysis Devices

**DOI:** 10.1021/acsami.3c10680

**Published:** 2023-10-09

**Authors:** Anetta Platek-Mielczarek, Johanna Lang, Feline Töpperwien, Dario Walde, Muriel Scherer, David P. Taylor, Thomas M. Schutzius

**Affiliations:** † Laboratory for Multiphase Thermofluidics and Surface Nanoengineering, Department of Mechanical and Process Engineering, ETH Zurich, Sonneggstrasse 3, Zurich CH-8092, Switzerland; ‡ Laboratory of Thermodynamics in Emerging Technologies, Department of Mechanical and Process Engineering, ETH Zurich, Sonneggstrasse 3, Zurich 8092, Switzerland; § Department of Mechanical Engineering, University of California, Berkeley, Berkeley, California 94720, United States

**Keywords:** reverse electrodialysis, RED, electrode rinse solution, ERS, blue energy, salinity gradient power, microfluidics, carbon electrodes, redox electrolyte

## Abstract

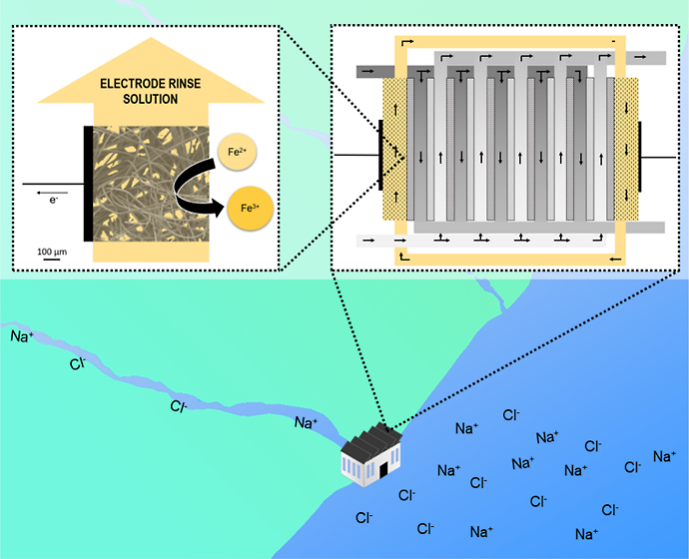

Natural salinity gradients are a promising source of so-called “blue energy”, a renewable energy source that utilizes the free energy of mixing for power generation. One promising blue energy technology that converts these salinity gradients directly into electricity is reverse electrodialysis (RED). Used at its full potential, it could provide a substantial portion of the world’s electricity consumption. Previous theoretical and experimental works have been done on optimizing RED devices, with the latter often focusing on precious and expensive metal electrodes. However, in order to rationally design and apply RED devices, we need to investigate all related transport phenomena—especially the fluidics of salinity gradient mixing and the redox electrolyte at various concentrations, which can have complex intertwined effects—in a fully functioning and scalable system. Here, guided by fundamental electrochemical and fluid dynamics theories, we work with an iron-based redox electrolyte with carbon electrodes in a RED device with tunable microfluidic environments and study the fundamental effects of electrolyte concentration and flow rate on the potential-driven redox activity and power output. We focus on optimizing the net power output, which is the difference between the gross power output generated by the RED device and the pumping power input, needed for salinity gradient mixing and redox electrolyte reactions. We find through this holistic approach that the electrolyte concentration in the electrode rinse solution is crucial for increasing the electrical current, while the pumping power input depends nonlinearly on the membrane separation distance. Finally, from this understanding, we designed a five cell-pair (CP) RED device that achieved a net power density of 224 mW m^–2^ CP^–1^, a 60% improvement compared to the nonoptimized case. This study highlights the importance of the electrode rinse solution fluidics and composition when rationally designing RED devices based on scalable carbon-based electrodes.

## Introduction

The global demand for energy requires constant deployment of renewable energy sources. However, one such source that is often overlooked is the natural mixing of fresh (ca. 0.012% NaCl) and saltwater (ca. 3.5% NaCl)^1,2^—like rivers flowing into the sea. The theoretical Gibbs free energy of mixing one cubic meter of fresh water with one cubic meter of saltwater gives 2 MJ, and if scaled globally has a yearly potential of 1000 TW h.^3,4^ Switzerland consumes yearly, on average, 225 TW h, according to the Federal Department of Foreign Affairs (FDFA).^5^ The two most important systems that can leverage this at the pilot-scale are pressure-retarded osmosis and reverse electrodialysis (RED).^6−8^ The former uses semipermeable membranes, which allow the fresh water to pass to the saltwater side through osmosis, building up significant pressures that can power turbines.^9^ The latter system uses alternating cation- and anion-exchange membranes (CEM and AEM, respectively), creating channels where on either side of each membrane freshwater and saltwater are passed.^10,11^ This arrangement allows salt ions to diffuse across the membranes in opposite directions^12,13^—due to the salinity gradient between the feed solutions—creating an electrical potential (Nernst) that drives reversible Faradaic reactions at the electrode/electrolyte interface^14^ and generates electricity.^3,15^ To facilitate this energy conversion process, an electrode rinse solution (ERS), consisting of two redox species—forming a redox pair—and a supporting electrolyte, is pumped, circulating it from one electrode to the other. The simplest RED system consists of one membrane, with freshwater and saltwater on either side, and this is defined as a cell pair (CP), which is in between two electrodes.^16,17^ In this way, the chemical potential between the feed solutions is directly fully converted into electrical energy, without being converted into thermal or mechanical energy.^18,19^ The RED device output voltage (*U*_max_) is directly proportional to the number of CPs (*n*) connected in series, and the typical number of CPs used in RED devices is *n* = 50,^20,21^ but applied pilot plant studies going beyond *n* = 100 are also reported.^22,23^ One of the main failure reasons of RED devices is membrane fouling, addressed in fundamental studies of different materials—improved ones significantly increase the overall cost of a final device.^24,25^

The most straightforward method to improve the power output of a real RED device is to modify its structure, especially membrane spacing defined as a membrane separation distance,^20,26−28^ the number of CPs,^29−31^ and the flow rate of the low- and high-salinity solutions.^30,32−34^ Typically, spacers are inserted between the anion- and cation-exchange membranes to form compartments filled with saline solutions.^14,30,33,35^ Nevertheless, depending on the properties of the spacers, they can induce more flow resistance and deteriorate the RED device performance.^36^ This is why spacer-less systems or modified textured membranes are also introduced.^9,29,37^ Various membranes’ modification and optimization for enhanced mass transport are reported.^38−40^ Several significant implications are outlined in theoretical predictions, investigating the impact of intermembrane distance on the power density of a RED system, but they remain unverified for these specific conditions.^41^ Universal findings discussed in the literature can be summarized as follows: the gross power density is higher for smaller membrane separation distances;^26^ the pressure drop, Δ*p*, across the device is strongly dependent on the membrane separation distance;^20,26^ the most common membrane separation distances are 100, 200, or 300 μm,^31,42,43^ which seem to achieve a good balance between the gross power output and the pumping power input; the use of mixing promoters can be beneficial for systems operating with laminar flows;^44^ textured membranes appear to be a better choice for decreasing the membrane separation distance than thin spacers (<100 μm);^20,27,44,45^ the membrane thickness should be as low as possible to achieve high gross power density;^46^ and the residence time of saline solutions—crucial for ensuring high ion flux through the membrane—can be tuned either by a membrane separation distance or feed solution flow rate.^10,43,46^ From this summary, it is clear that work has been done on optimizing the gross power output from RED devices for a given pumping power input—the difference between the two being the net power output—by focusing on tuning the fluidics between the membranes.^47^ However, what has been neglected in these studies is the pumping power needed to circulate the ERS and its relationship to the net power output, which is nontrivial, taking into account both losses as well as the ability to transport redox species to the electrode interfaces driving the necessary reactions.

The gross power output from a RED device can also be increased by engineering the composition and concentration of the ERS and the composition and texture of the electrode interface, preferably sustainable and scalable.^48−50^ The lifecycle of materials used in the energy storage/conversion systems and their environmental impact are of high importance;^51−53^ therefore, carbon attracts scientists’ attention in various electrochemical applications.^48,54−57^ Previous works have used carbon-based and metallic electrodes in RED devices: the former being carbon felt that is compatible with more benign redox electrolytes^58,59^ and the latter being platinum, titanium, ruthenium, or iridium for their catalytic activity.^14,34,44,60,61^ For carbon-based electrodes, previous works have used a reversible iron-based redox pair, Fe^2+^/Fe^3+^ (concentration ranges from 20 to 50 mM),^58,62^ with the supporting electrolyte NaCl or Na_2_SO_4_.^63^ For metallic electrodes, which have been studied to a higher extent than their carbon-based counterparts, many RED devices use Fe(CN)_6_^3+^/Fe(CN)_6_^4+^ as the redox pair with a range of concentrations from 50 to 400 mM,^27,34,35,64−66^ with the supporting electrolyte NaCl. In each of these studies, regardless of the chosen electrode material and ERS, various ERS flow conditions have been implemented. Depending on the electrode size and texture, these ERS flow rates have ranged from 0.03 to 30 L min^–1^. D’Angelo et al. studied the effect of the ERS flow rate on the gross power output,^67^ and Tedesco et al. scaled up a RED device considering all circulating solutions (freshwater, saltwater, and ERS).^68,69^ From this, we see that a range of redox species concentrations and ERS flow rates have been investigated;^70^ however, a RED device performance for net power output was not optimized. We find that a holistic view on a RED device net power output, accounting for the pumping power density input of the saline solutions and ERS for a range of redox species concentrations, is missing, especially for carbon-based electrodes.

Here, guided by fluid dynamics and electrochemical theories, we systematically investigate the effects of saline and ERS fluidics on gross and net power densities from a RED device. To illustrate this approach, we use carbon-based electrodes, which are scalable, cheap, and compatible with benign redox species, Fe^2+^/Fe^3+^. First, we explore experimentally the effect of engineering the membrane separation distance together with implementing ERS in the RED stack. We report a gross power density and a pumping power density, resulting in a net power density output, which provides a clear evaluation of the proposed systems. Guided by fundamental electrochemical characterization, we discuss the ERS composition and its further performance in a RED system. We explain the physics and chemistry behind RED operation by exploring the effect of electrolyte optimization, i.e., the concentration of the redox pair and supporting electrolyte, flow conditions, and fluidics engineering, to enhance energy conversion. Finally, to show the promise of this approach, we demonstrate a net power density of 224 mW m^–2^ CP^–1^ for a *n* = 5 CP RED device, a 60% improvement compared to the nonoptimized system defined as a control case. These results are underpinned by comparison of the fluidic and adsorption-reaction time scales.

## Results and Discussion

**Figure 1:**
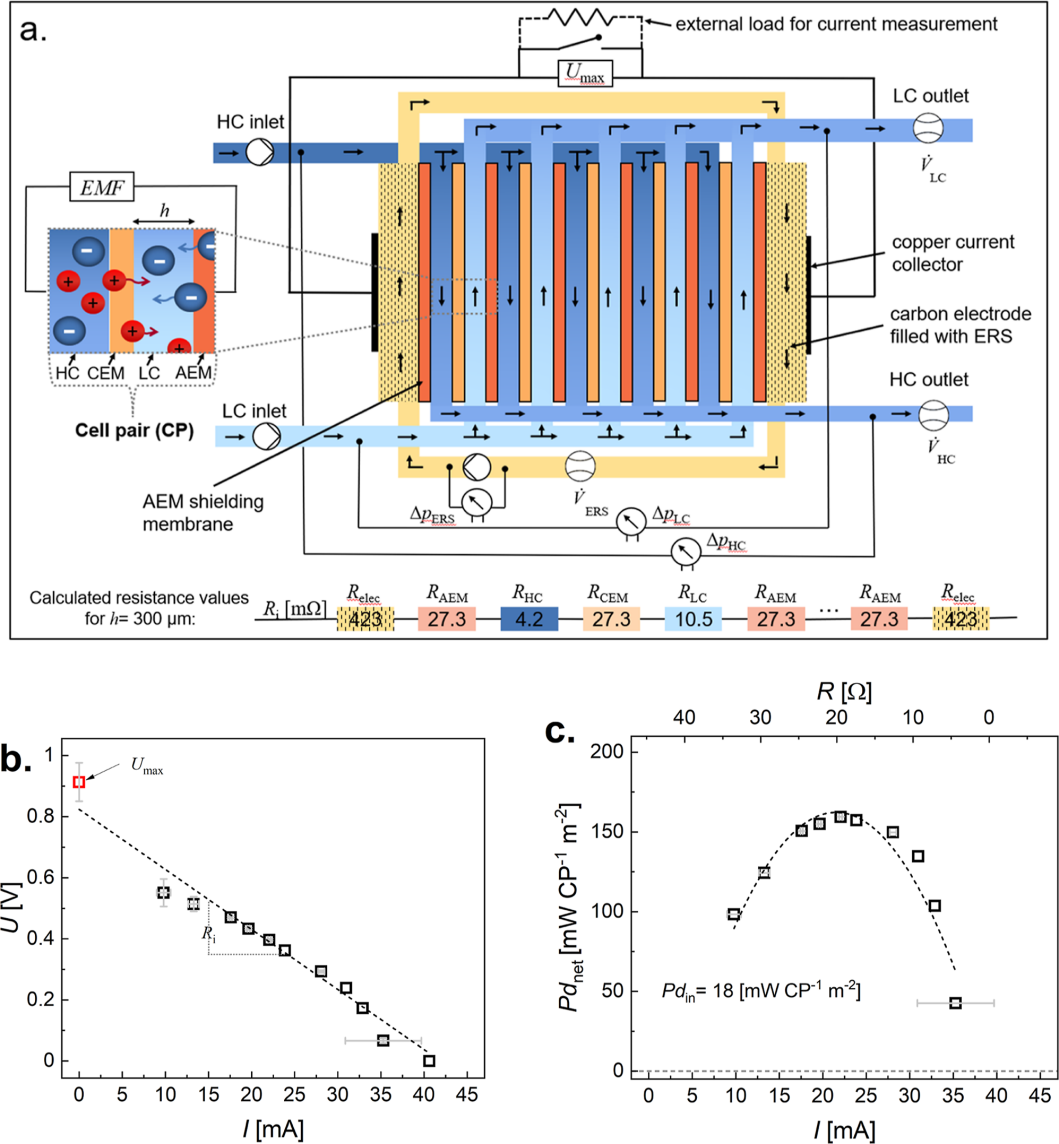
RED device design and characterization. (a) Schematic showing the RED device, which consists of alternating CEM and AEM separated by a distance, *h*, and filled with LC and HC salinity water solutions, which are pumped in between the membranes. An individual membrane–water contact area for one side of the membrane is defined as *A*. Each pair of CEM and AEM with an LC and a HC compartment in between forms a “cell pair” (CP) that creates Nernst potential called the electromotive force, EMF, and this device consists of *n* = 5 CPs what gives 5EMF voltage output. Each end of the device is connected to a carbon electrode inside a compartment where the ERS is pumped and circulated through both carbon electrodes. The electrodes are connected to a copper current collector, which is connected to an external load for indirect current, *I*, measurements by closing the circuit with selected resistors by reading the voltage, *U*. We can control and measure the flow rate of the HC, LC, and ERS solutions as well as the pressure drop along the HC and LC flow paths and the pressure drop across the ERS pump. Electrical resistance values of various device components, *R*_*i*_, are also shown. These values are calculated using the following assumptions: *h* = 300 μm, HC = 1 M NaCl, LC = 0.01 M NaCl, ERS = 20 mM FeCl_2_ = FeCl_3_ in 0.01 M NaCl and carbon-felt electrodes (see Supporting Information Note 1). Performance of the RED device with *n* = 5 at a fixed flow rate of saline solutions (0.21 cm^3^ s^–1^) and ERS (0.05 cm^3^ s^–1^) using external load resistors: (b) Voltage, *U*, vs current, *I* and (c) net power density, *Pd*_net_ = *UI*/(*An*), vs *I* and vs external load resistance, *R*, with a fixed pumping power density, *Pd*_in_= (Δ*p*_LC_*V̇*_LC_ + Δ*p*_HC_*V̇*_HC_ + Δ*p*_ERS_*V̇*_ERS_)/(*nA*). This is the configuration used throughout the study, where individual parameters are varied, but *n* and *A* are kept constant. Error bars are calculated standard deviations of at least three independent RED device experimental runs, some of them are within the experimental scatter point. On each *Pd*_net_ figure presented in this manuscript with standard deviation error bars, *Pd*_in_ values are also plotted.

The RED device used in this study is built with *n* = 5 CPs connected in series with carbon-felt electrodes at either end of the device. Each CP consists of a CEM and an AEM separated by a distance *h*. Placing the CPs in series forms compartments where the low concentration (LC; 0.01 M NaCl) and high concentration (HC; 1 M NaCl) salinity solutions are pumped through. The values of *h* are varied between *h* = 100, 125, and 300 μm. A schematic of the RED device is presented in Figure 1a with the calculated electrical resistance values, *R*, for each component (assuming *h* = 300 μm; see Supporting Information Note 1). The concentration difference between the feed solutions is what generates the Nernst potential across the membrane (Supporting Information Note 2). When feed solutions flow through the intermembrane compartments, the voltage spontaneously builds up depending on the membranes’ permselectivity. Finally, an ERS, consisting of Fe^2+^/Fe^3+^ redox species, is circulated over the electrodes, performing reversible reactions and generating current that can power external devices.

To measure the power produced by the RED device, the circuit was closed using resistors with variable resistance, and the voltage drop over the resistor was measured, enabling the calculation of steady-state current output, *I*, from the device, Figure 1b. In order to compare different devices, the operational parameter—power density, *Pd*, should be reported, which depends on the number of CPs, *n*, and the area of the membrane/saline solution interface, *A*. The gross power density out of the device is therefore *Pd*_gross_ = *IU*/(*nA*) (see Supporting Information Note 3). However, this parameter does not account for the power density needed to pump the saline solutions and ERS, *Pd*_in_= (Δ*p*_LC_*V̇*_LC_ + Δ*p*_HC_*V̇*_HC_ + Δ*p*_ERS_*V̇*_ERS_)/(*nA*), where Δ*p* and *V̇* are the pressure difference and volumetric flow rates, respectively, across the HC, LC, and ERS flow paths. Differential pressure sensors are introduced at the inlet and the outlet of the LC and HC flow paths, and Δ*p*_ERS_ is measured with a pressure sensor across the pump that is circulating the ERS. With this, one can define the net power density, *Pd*_net_ = *Pd*_gross_ – *Pd*_in_, Figure 1c. The maximum *Pd*_net_ recorded for the current configuration, which is based on the conditions most commonly used in RED studies,^10,26,30^ is 159 mW CP^–1^ m^–2^ (LC: 0.01 M NaCl with *V̇*_LC_ = 0.21 cm^3^ s^–1^; HC: 1 M NaCl with *V̇*_HC_ = 0.21 cm^3^ s^–1^; ERS: 20 mM Fe^2+^ = Fe^3+^ in 0.01 M NaCl with *V̇*_ERS_ = 0.05 cm^3^ s^–1^, *n* = 5, *A* = 110 cm^2^).

**Figure 2:**
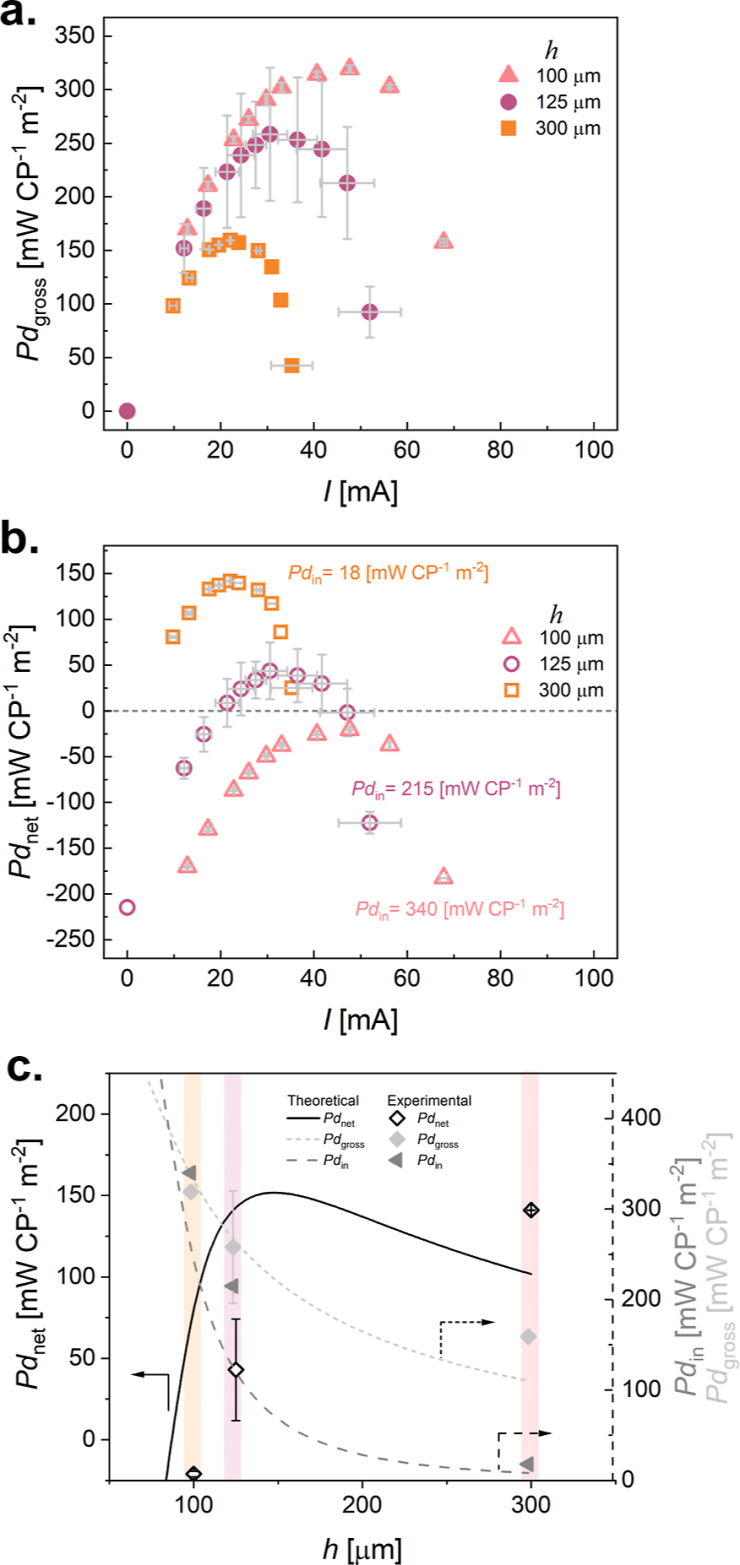
Optimizing the net power density output through fluidic engineering. RED device, *n* = 5, with varied *h*, *h* = 100, 125, and 300 μm: (a) *Pd*_gross_ vs *I* and (b) *Pd*_net_ vs *I*. Fixed flow rates for the saline solutions (*V̇*_LC_ = *V̇*_HC_ = 0.21 cm^3^ s^–1^) and the ERS, 20 mM FeCl_2_ = FeCl_3_ in 0.01 M NaCl (*V̇*_ERS_ = 0.05 cm^3^ s^–1^). When operating the RED device for constant flow rates of saline solutions and ERS, for small *h* (100 μm), *I* increases together with the pumping power *Pd*_in_ (LC, HC, and ERS), leading to negative *Pd*_net_. Optimum RED stack design with *n*= 5 for different channel heights, *h*, e.g., membrane separation distance, using LC–0.01 M NaCl and HC–1 M NaCl solutions: (c) maximal gross power density, *Pd*_gross_, and pumping power, *Pd*_in_, where the data points are experimental measurements (each data point represents an average of at least 3 experiments), and the lines are theoretical: *Pd*_gross_ based on a semiempirical assumption of *U*_max_ and *I*_max_, resulting in a trend *Pd*_gross_ = B/*h*; *Pd*_in_ is computed based on the flow rate and the pressure drop (described above)—*Pd*_in_ = Σ*V̇*_*i*_Δ*p*_*i*_*,* where *i* counts for LC, HC, and ERS, following the correlation *Pd*_in_ = A/*h*^3^.

Various membrane separation distances *h* were tested in order to find the optimal configuration for further tests in terms of *Pd*_net_, *h* = 100, 125, and 300 μm, Figure 2. The recorded *U*_max_ values for those configurations are comparable, ca. 0.9 V (see Figure S1); however, *I* depends on *h*. This correlation is almost proportional, with three times decrease in the channel height (*h* = 300 μm to *h* = 100 μm) resulting in a 2-fold increase of *Pd*_gross_: 159 mW CP^–1^ m^–2^ to 319 mW CP^–1^ m^–2^. This is mainly caused by manipulating the resistance of the LC compartment, *R*_LC_, by changing the *h*. Interestingly, if a similar correlation is done for *Pd*_net_, the opposite trend is observed. The RED device with *h* = 100 μm exhibits the highest *Pd*_gross_ but is not capable of producing enough power to maintain its own operation, *Pd*_net_ < 0. This is the result of the high pumping power requirements and a high value of *Pd*_in_. Figure 2c shows a plot of experimental data of maximum *Pd*_net_, *Pd*_in_, and *Pd*_gross_ for *h* = 100 μm, 125 μm, and 300 μm and their estimated values. We assume that *Pd*_in_ is proportional to A/*h*^3^, a Hagen–Pouiseulle flow, and is justified on the basis of the Reynolds number for all experiments conducted, *Re* < 1 (see also Supporting Information Note 4 and Figure S2 for the relationship between the flow rate and the pressure drop). We assume that *Pd*_gross_ is proportional to B/*h*, attributed to gradients in salinity concentration within the liquid, and following a Fickian diffusion process normal to the flow direction. Assuming these forms, a best fit is performed on the experimental data with *Pd*_net_ = *B*/*h* – *A*/*h*^3^ (with constant values *A* = 2.25 × 10^8^; *B* = 33,030). From this, we see that by only analyzing the fluidics between the membranes, and using this simplified analysis, one can expect that we are operating close to the optimum condition (*h* = 150 μm). Based on what has been proposed in the literature,^71^ passive mixers’ influence on *Pd*_net_ was verified for *h* = 300 μm; however, we did not observe any improvements for our RED device (see Supporting Information Note 5 and Figure S3). Thus, we found *h* = 300 μm as our optimal configuration with respect to the fluidic design, for which *Pd*_net_ exceeds the theoretical prediction; further improvements will be done at these conditions. Next, the focus was on engineering the electrode/electrolyte interface to increase *I*, and, in effect, boost the *Pd*_net_ of the RED device.

**Figure 3:**
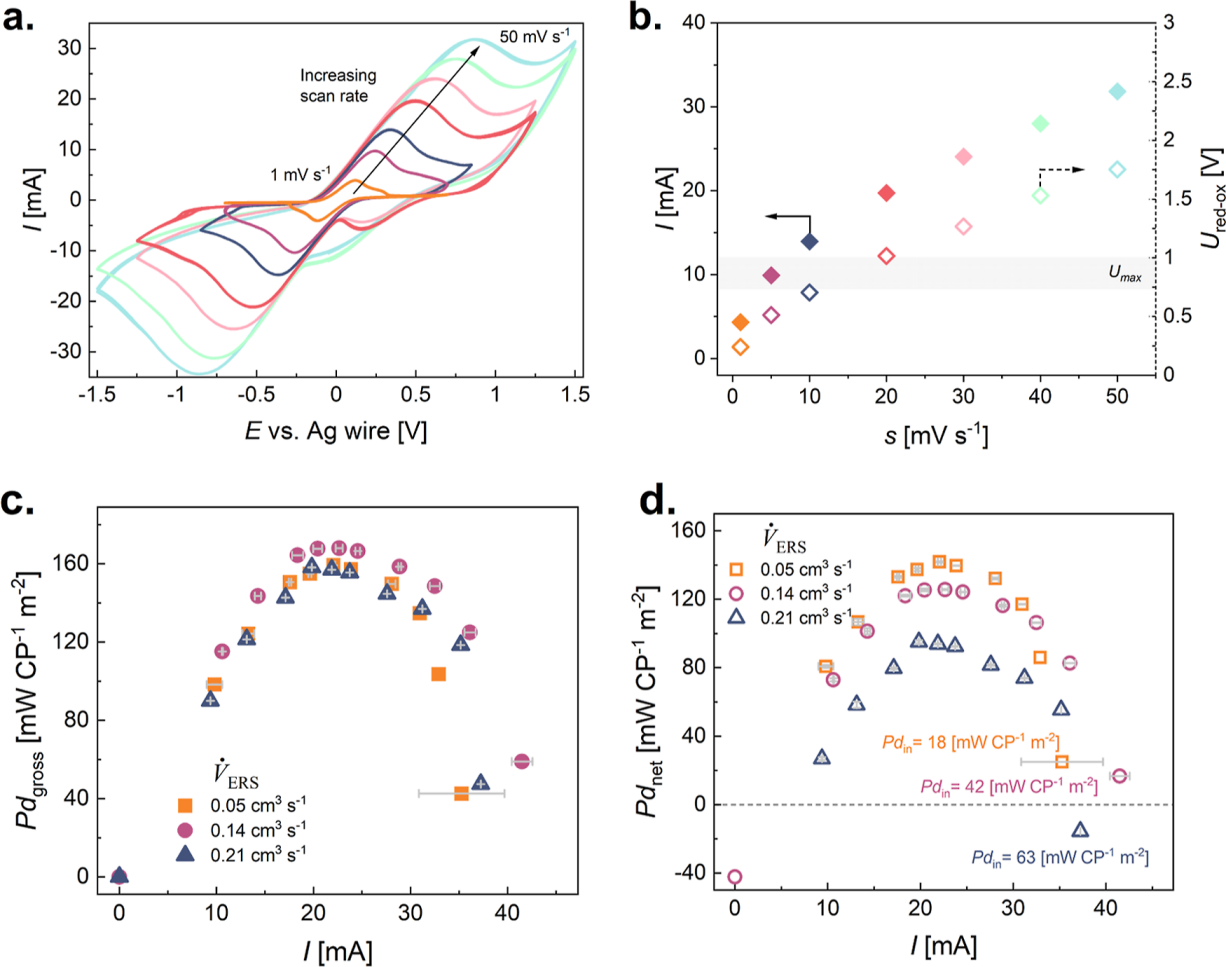
Engineering the ERS fluidics for enhancing the net power output. Electrochemical characterization of redox electrolyte solution with composition 20 mM FeCl_2_ = FeCl_3_, 0.01 M NaCl: (a) cyclic voltammetry at various scan rates (1, 5, 10, 20, 30, 40, and 50 mV s^–1^) in the three-electrode setup with Ag wire as a quasi-reference electrode. Potential window is adjusted to reversible oxidation and reduction redox reaction peak, *U*_red-ox_; (b) maximum current peak (*I*) and potential difference (*U*_red-ox_) of Fe^2+^/Fe^3+^ redox reaction vs scan rate with *U*_max_ of RED device with *n* = 5. (c) Performance of RED device with *n* = 5 using LC and HC at flow rates *V̇*_HC_ = *V̇*_LC_ = 0.21 cm^3^ s^–1^ in *h* = 300 μm channels with ERS in the carbon electrode compartment (20 mM FeCl_2_ = FeCl_3_, 0.01 M NaCl) at different flow conditions resembling 2, 5, and 10 mV s^–1^ (see Supporting Information Note 6): *Pd*_gross_ vs *I*; (d) *Pd*_net_ vs *I* with pumping power values (LC + HC + ERS).

For carbon-based electrodes, a sustainable Fe^2+^/Fe^3+^ redox pair has been proposed.^58,62^ However, considering the ERS composition and flow conditions, it has not been elaborated in the RED device. Previous work has used the following ERS composition with metallic electrodes: 20 mM Fe^2+^ = Fe^3+^.^58,67,69^ Therefore, we select this specific composition and study the effect of flow rate to identify an optimum based on *Pd*_net_, Figure 3. First, the ERS is characterized fundamentally outside of the RED device (see Methods) by applying different scan rates *s* = 1 mV s^–1^ to *s* = 50 mV s^–1^, Figure 3a. The maximum redox peak *I*_max_ increases with increasing *s*, according to the charge preservation theory.^72^ What needs to be considered is the *U*_max_ of the RED device, which is strictly correlated with *n*. Therefore, not all *s* would be applicable in the real RED device, as the limiting factor is its maximum voltage—the potential difference between the oxidation–reduction reactions, *U*_red-ox_, should be within *U*_max_. From Figure 3b, one can see that in the case of *n* = 5, only 2, 5, and 10 mV s^–1^ can be applied in a way that *U*_red-ox_ ≤ *U*_max_. The residence time of an ERS within a carbon electrode, τ, can be calculated based on τ = *U*_red-ox_/*s* (see Supporting Information Note 6). Then, knowing our electrode volume, *V* = 28 cm^3^, and the time that ERS should be spent within its bulk for selected scan rate *s* = 2 mV s^–1^, *s* = 5 mV s^–1^, and *s* = 10 mV s^–1^, τ = *U*_red-ox_/*s*, we can calculate *V̇*_ERS_ = *V*/τ, and we can correlate it with the *s* that was applied in fundamental cyclic voltammetry tests. We therefore tested various *V̇*_ERS_ near this estimated value of *V*/τ = 0.28 cm^3^ s^–1^ that was calculated for *s* = 10 mV s^–1^, so 0.21 cm^3^ s^–1^, *s* = 5 mV s^–1^: 0.14 cm^3^ s^–1^, and *s* = 2 mV s^–1^: 0.05 cm^3^ s^–1^. The scan rate *s* of 10 mV s^–1^ is limited not only by *U*_red-ox_ for the RED with *n* = 5 but also by the pumping system’s upper limitations (lower limitation of the applied flow rate is 0.05 cm^3^ s^–1^). The highest flow rate, *V̇*_ERS_ = 0.21 cm^3^ s^–1^, is predicted to be the best among all ERS flow rates tested in the RED device, according to the high current recorded in the fundamental studies. From Figure 3c, it is clear that *Pd*_gross_ is not affected by the flow rate, indicating that we are in some type of adsorption-reaction-limited regime. Therefore, further increasing the flow rate should increase *Pd*_in_ and result in a decrease in *Pd*_net_, which is seen in Figure 3d. In contrast to metallic electrodes, when carbon-based electrodes are flushed with ERS, no increase in *Pd*_gross_ is observed (because of overlapping *U–I* curves with increasing *V̇*_ERS_—Figure S4). This clearly indicates that such an electrode/electrolyte system is adsorption-reaction-limited, as the time scale for Fe^2+^ or Fe^3+^ to diffuse toward redox-active sites at the carbon interface is much longer^73^ than the one obtained with the lowest *V̇*_ERS_. Based on the diffusion coefficient of Fe^2+^, Fe^3+^ in water, *D*_Fe^2+^_ = 7.19 × 10^–6^ cm^2^ s^–1^, *D*_Fe^3+^_ = 6.04 × 10^–6^ cm^2^ s^–1^, and electrode thickness, 0.25 cm, the average diffusion time equals 49,000 s—ca. 13.5 h. The electrolyte solution can search for easy passages within carbon felt electrodes to pass through, as it has substantial electric resistance (423 mΩ—Figure 1). When metal electrodes are in use, no limitations are observed—diffusion and adsorption, reaction—and the flow rate of ERS directly increases the recorded current.^67^ Therefore, engineering of carbon-based electrodes should be the focus of future research to enhance wetting and ERS passages through porous electrode’s bulk. Thus, to optimize *Pd*_net_ of a RED device with *n* = 5 with carbon-based electrodes, slow *V̇*_ERS_ are recommended, and in our study *V̇*_ERS_ = 0.05 cm^3^ s^–1^ was further used.

**Figure 4:**
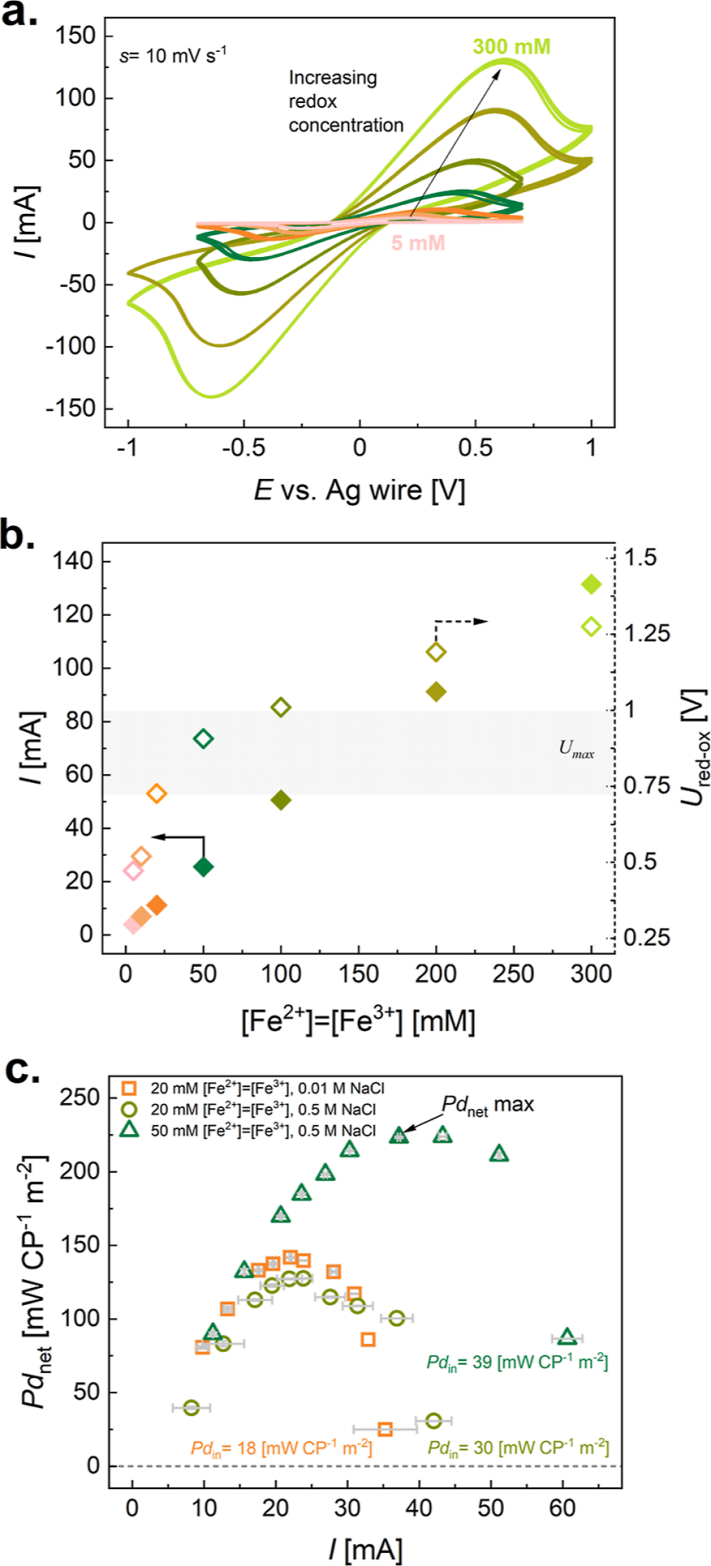
Rationalization of the ERS composition for carbon electrodes, guided by fundamental studies: redox pair and supporting electrolyte concentration in the specific RED device (*n* = 5). Electrochemical characterization of the redox pair (Fe^2+^/Fe^3+^) and the supporting electrolyte (NaCl) with various concentrations: (a) cyclic voltammetry at 10 mV s^–1^ for concentrations from 50 to 300 mM of redox species with 0.01 M NaCl; (b) maximum current peak (*I*) and potential difference (*U*_red-ox_) of the Fe^2+^/Fe^3+^ redox reaction vs redox pair concentration. Performance of the RED device with three optimized compositions of the ERS, with *n* = 5, *h* = 300 μm, *V̇*_LC, HC_ = 0.21 cm^3^ s^–1^, and *V̇*_ERS_ = 0.05 cm^3^ s^–1^: (c) *Pd*_net_ vs *I* with pumping power values.

After selection of the most optimal ERS flow conditions for the RED device with *n* = 5 and carbon-based electrodes, we focused on the optimization of ERS composition—concentration of redox species, Fe^2+^/Fe^3+^, Figure 4, and the supporting electrolyte, NaCl. The initial supporting electrolyte concentration equals LC—0.01 M—to avoid any cross contamination of ERS in the LC compartment. Thus, we also optimize the device’s performance with respect to the supporting electrolyte concentration (Figure S5). From fundamental studies of the scan rate on the redox activity of the Fe^2+^/Fe^3+^ redox pair, the highest *s* that could have been applied in the RED device (within the electrode compartment) is 10 mV s^–1^. Thus, cyclic voltammetry with *s* = 10 mV s^–1^ is conducted for ERS with Fe^2+^/Fe^3+^ concentrations from 5 to 300 mM, the concentrations that are shown in previous studies of RED devices.^58,62,67,69^ With the evaluation of *U*_red-ox_ and *I*, one can see that only a concentration up to 50 mM lies in the *U*_max_ region of the RED device with *n* = 5 and *h* = 300 μm. This is why we find it important to rationalize selected conditions for application of ERS within the RED device to fully exploit its power generating potential. By increasing the supporting electrolyte concentration, a delicate difference in the *U*–*I* curves can be observed for 20 mM Fe^2+^=Fe^3+^. However, considering the increase of redox species concentration, one can notice a big improvement of *Pd*_gross_ (see Figure S5) and *Pd*_net_. Flow rates of LC, HC, and ERS are the same for each experiment; therefore, an increase of *Pd*_net_ results directly from increased *I* for ERS with 50 mM Fe^2+^=Fe^3+^, reaching a maximum value of 224 mW CP^–1^ m^–2^, Figure 4c. This indicates that, by applying a mesoporous carbon-based electrode, one can tune *Pd*_net_ by applying more concentrated ERS. The optimal concentration is correlated with the number of CPs, *n*, in the RED device—thus, both factors should always be considered and tested together. We prove that for sustainable carbon electrode/ERS interface—with FeCl_2_ and FeCl_3_—one can easily manipulate *Pd*_net_ by increasing the concentration of redox species in accordance with *U*_max_ of RED device related to *n* while keeping a low *Pd*_in_ penalty for pumping the ERS. Even if the pumping power used for ERS circulation is lower than those for LC and HC, it should not be neglected. Thus, our work has been compared with state-of-the-art publications in Table 1, highlighting various in-house experimental conditions and RED performance metrics obtained for device laboratory-scale studies.

**Table 1:** Selected RED Devices Described in the Literature Based on Carbon Derivatives and Compared to Precious Metal-Based Electrode Systems^a^

electrode material	*n*	LC	*V̇*_LC_	HC	*V̇*_HC_	ERS	*V̇*_ERS_	*Pd*_in_=	*Pd*_gross_max_	*Pd*_net_max_	refs
carbon-based	5	0.01 M	0.21 cm^3^ s^–1^	1 M	0.21 cm^3^ s^–1^	50 mM FeCl_2_	0.05 cm^3^ s^–1^	Pd_inLC_ + Pd_inHC_ + Pd_inERS_ = 39 mW CP^–1^ m^–2^	263 mW CP^–1^ m^–2^ = 1.32 W m^–2^	224 mW CP^–1^ m^–2^*=* 1.12 W m^–2^	this work
						50 mM FeCl_3_					
						0.5 M NaCl					
	50	*0.017 M*		*0.5 M*		*50 mM FeCl*_2_					(62)
						*50 mM FeCl*_3_					
						0.5 M NaCl–HCl					
	100	tap water	1.66 cm^3^ s^–1^	natural seawater	*1.66 cm*^3^*s*^*–1*^	NaCl	*1.66 cm*^3^*s*^*–1*^		0.72 W m^–2^		(77)
	2	0.017 M	*1.66 cm*^3^*s*^*–1*^	0.6 M	*1.66 cm*^3^*s*^*–1*^	50 mM Fe(SO_3_)	*5 cm*^3^*s*^*–1*^		1.34 W m^–2^		(63)
						50 mM Fe_2_(SO_3_)_3_					
						*1.2 M NaCl*					
	29	*0.017 M*	*1.66 cm*^3^*s*^*–1*^	*0.6 M*	*0.66 cm*^3^*s*^*–1*^	NaCl			259 mW CP^–1^ m^–2^= *7.5* W m^*–2*^		(78)
precious metal-based	25	0.02 M	*12.5 cm*^3^*s*^*–1*^	4 M	*12.5 cm*^3^*s*^*–1*^	0.1 MK_3_Fe(CN)_6_			4.78 W m^–2^		(27)
						0.1 MK_4_Fe(CN)_6_					
						2 M NaCl					
	5	1.6	5 cm s^–1^	5 M	3 cm s^–1^	0.1 M K_3_Fe(CN)_6_	1 cm s^–1^	Pd_inLC_ + Pd_inHC_	10.5 W m^–2^		(66)
						0.1 M K_4_Fe(CN)_6_					
						0.6 M NaCl					
	50	0.1 M	1 cm s^–1^	5 M	1 cm s^–1^	0.1 M K_3_Fe(CN)_6_	30 L h^–1^*= 8.33 cm*^3^*s*^*–1*^	Pd_inLC_ + Pd_inHC_		4.5 W m^–2^	(64)
						0.1 M K_4_Fe(CN)_6_					
						2.5 M NaCl					
	50	1 g L^–1^*= 0.017 M*		30 g L^–1^ = *0.5 M*		50 mM K_4_Fe(CN)_6_		Pd_inLC_ + Pd_inHC_		0.71 W m^–2^	(10)
						50 mM _3_Fe(CN)_6_					
						0.25 M NaCl					
	50	1 g L^–1^*= 0.017 M*	0.4 L min^–1^ = *6.7 cm*^3^*s*^*–1*^	30 g L^–1^*= 0.5 M*	0.4 L min^–1^ = *6.7 cm*^3^*s*^*–1*^	50 mM K_4_Fe(CN)_6_	60 mL min^–1^*= 1 cm*^3^*s*^*–1*^	1 *Pd*_gross_	0.93 W m^–2^		(21)
						50 mM K_3_Fe(CN)_6_					
						1 M NaCl					

a In *italics* are highlighted values recalculated from ref. to the same unit as presented within this work.

## Conclusions

Our investigation of the RED device with five CPs reveals that fluidic engineering (such as height and mixing conditions) does not offer significant room for further improvement when commercially available membranes and carbon-based electrodes are used. However, we discovered that the composition and flow conditions of the ERS, which are often overlooked in RED studies, can significantly affect the overall RED performance and should not be ignored. By conducting fundamental three-electrode studies of the electrochemical behavior of the ERS, we were able to preselect the composition and operational conditions that led to the optimization of the RED device using three different electrolytes (vary in the concentration of redox species and the supporting electrolyte). We show that rational selection of ERS and its flow conditions can affect the maximum current and redox reaction voltage difference, which need to be adjusted to the system’s operational metrics, specifically, the maximum voltage of the RED device. Considering it, *Pd*_net_ can be increased by keeping a low *Pd*_in_ penalty for the ERS flow and a high redox activity by using redox species with higher concentrations.

## Methods

### RED Device

The in-house-made RED stack consists of two PLEXIGLAS end plates, in between which the electrodes—filled with ERS, membranes, and fluid compartments of LC and HC solutions (channels formed by gaskets—a membrane separation distance) are pressed together with 10 screws. Each end plate has an inlet and an outlet for the ERS. The front end plate has inlets for LC and HC saline solutions in the counter-flow arrangement, and the back end plate has the corresponding outlets. In the electrode compartment, adhesive copper foil playing the role of a current collector is directly connected to a carbon felt electrode (SGL Carbon GFD 2.5 EA) and connected to external wiring. ERS co-flows—within the carbon electrode’s bulk—with the neighboring LC and HC solutions. To avoid ionic short-circuiting, the length of the connecting tube between the two electrodes (ERS must pass one over each other before making the closed loop) is chosen to be 65 cm long with a tubing resistance of 142 MΩ. As the tubing resistance is higher by an order of magnitude than *R*_i_—the internal resistance of the RED device—no short-circuit losses are expected.^74^ Commercial ion-separation membranes are selected: AEM FAS-PET-75—anion-exchange membrane and CEM FKS-PET-75—cation-exchange membrane (Fumasep) with a thickness of 75 μm. Membranes tend to expand when in contact with water, so they are presoaked in DI water for 72 h, and later holes for the screws are punched. Three different materials are utilized as the gasket—NBR with a thickness of *h* = 300 μm, MDT foil (3M) with *h* = 125 μm, and Folex foil (PE) with *h* = 100 μm. No additional spacers are used. MDT foil is a three-layer adhesive tape that after operation within a RED device tend to self-detach its layers. Thus, we believe that its low mechanical integrity causes big experimental deviations for RED device runs with *h* = 125 μm visible in the biggest error bars. Passive micromixers are laser cut (Trotec Laser Cutter, Speedy 300) from Folex foil using the following parameters: power, 10; velocity, 1%; and frequency, 1 kHz. To reconstruct the *h* = 300 μm channel, either one NBR gasket or three Folex foils, F–F–F, are placed between two membranes.

### Redox Electrolyte

FeCl_2_, FeCl_3_, and NaCl are purchased from Sigma-Aldrich with ACS quality min. 95%. DI water is used to prepare all solutions. ERS is always done in the same order: dissolution of NaCl, FeCl_3_, and FeCl_2_ in appropriate mass defined by the molar concentration of the final solution. For one experimental run of a RED operation, a loop of 250 mL of ERS is employed for a RED stack with *n* = 5 and *A* = 110 cm^2^. ERS is prepared freshly once per week and kept without light exposure to avoid any side reactions or decomposition of iron-based species. ERS composition optimization is done and verified within a RED device, *n* = 5 μm and *h* = 300 μm, for three formulations
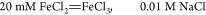
i
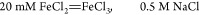
ii
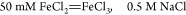
iii

### Conductivity and pH Tests

LC and HC solutions are verified before and after operation using electrochemical impedance spectroscopy at 100 kHz in a Teflon ring (10 mm in diameter and 4.4 mm in height) placed in a Swagelok Teflon body with stainless-steel current collectors, Table 2. The extrapolated real part of the impedance, *Z*(*Re*), where the imaginary *Z*′(*Re*) = 0, is used for conductivity calculations, that is, σ = 1/*Z*(*Re*).

**Table 2:** Physicochemical Characterization of Saline Solutions (HC and LC) and Redox Electrolyte (ERS: 20 mM FeCl_2_ = FeCl_3_ in 0.01 M NaCl) before and after Operation of the RED Device with *n* = 5, *h* = 300 μm, *V̇*_HC_ = *V̇*_LC_ = 0.21 cm^3^ s^–1^, *V̇*_ERS_ = 0.05 cm^3^ s^–1^, at the Inlet and Outlet

	inlet	outlet
aqueous solutions	σ (mS cm^–1^)	σ (mS cm^–1^)
HC	87	44
LC	1	53
ERS	14	10

### Characterization of the Redox Electrolyte

**Figure 5:**
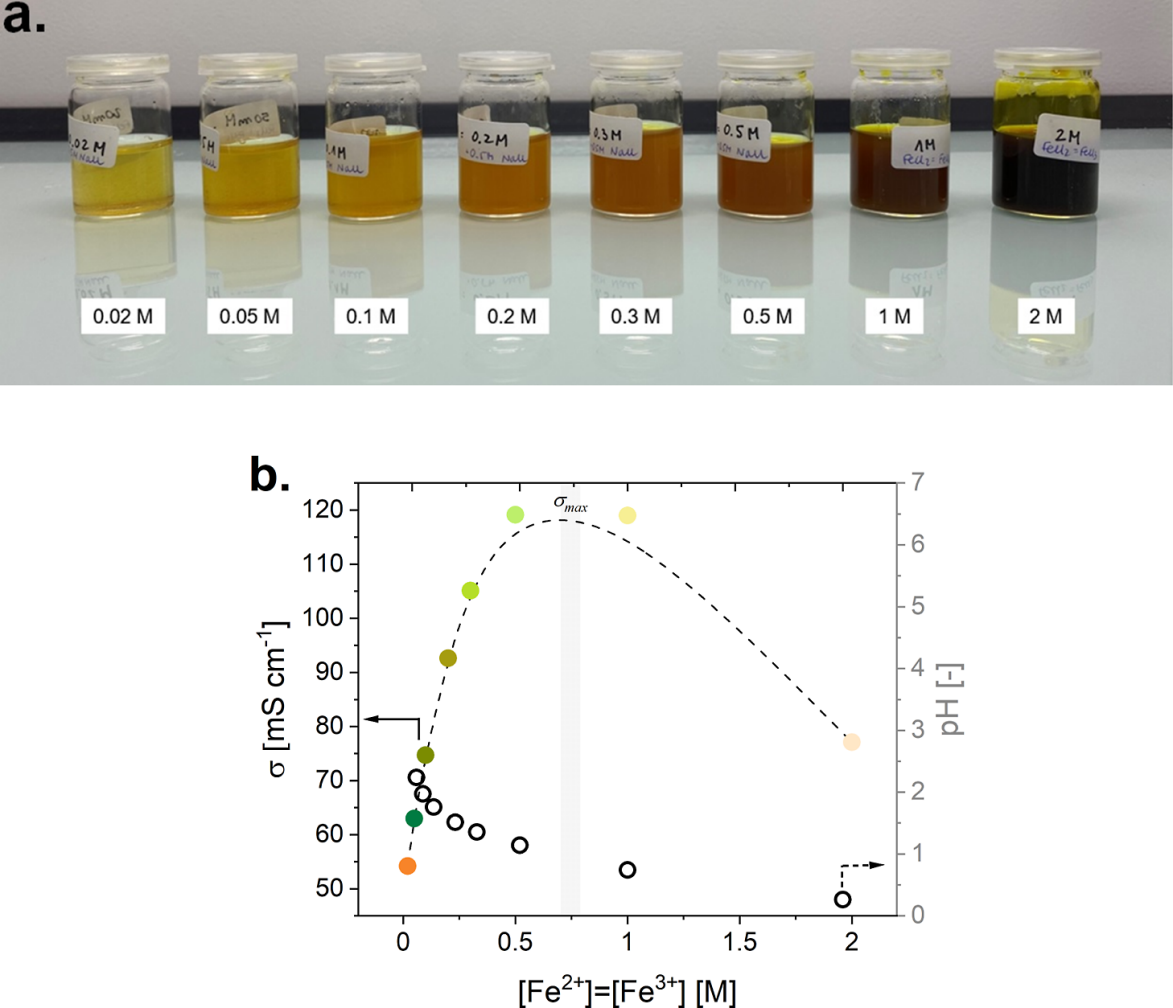
Physico-chemical characterization of ERS with Fe^2+^ = Fe^3+^ = 0.02, 0.05, 0.1, 0.2, 0.3, 0.5, 1, and 2 M in 0.5 M NaCl supporting electrolyte: (a) photo of ERS solutions (∼20 mL); (b) conductivity and pH vs concentration of the Fe^2+^/Fe^3+^ redox pair.

Fundamental characterization of the ERS is done in a three-electrode setup with an excess of electrolyte using the cyclic voltammetry technique (with scan rates ranging from 1 to 50 mV s^–1^). All measurements (at different scan rates, ERS concentrations, and ERS compositions) are done using a potentiostat/galvanostat SP-200 (Biologic). 20 mL of solution is tested using two carbon felt electrodes (counter electrode twice the size of the working one, 2 × 112 to 2 × 56 mm^2^), and a Ag wire is employed as a quasi-reference electrode, after verifying its stable potential in studied solutions. All ERS solutions have pH < 3, as shown in Figure 5. According to the Pourbaix diagram of iron species in aqueous solutions,^75^ the potential stability of the Fe^2+^/Fe^3+^ redox pair is constant for pH < 3.

The highest conductivity of ERS with 0.5 M NaCl supporting electrolyte has been found for a concentration of Fe^2+^/Fe^3+^ redox pair equal to 0.75 M, Figure 5. After exceeding this concentration, deterioration of the ERS performance, especially its reversibility, is expected. Such behavior is in accordance with physicochemical properties of aqueous electrolyte solutions.^76^

### Characterization of the RED Device

After building the RED device with *n* = 5, all of the fluid compartments are flushed with DI water for 5 min to get rid of trapped air bubbles and possible contamination. ERS is first connected to the RED stack, and electrode compartments are flushed with it as long as ERS is collected at the outlet (observed in the visible yellow color of ERS solution compared to colorless DI water), usually for an additional 5–10 min. This way, electrodes are fully filled with the redox electrolyte before connecting the LC and HC saline solutions. After the ERS is reaching the outlet of the second electrode, the ERS outlet is connected to the first electrode inlet reservoir preserving a closed ERS loop. From this point, the voltage is recorded until reaching the plateau of *U*_max_ using a Keithley 2000 multimeter. After *U*_max_ is reached and stabilized (ca. 1 V), the voltage drop across a series of external load resistors is measured, namely, 1.88, 5.26, 7.72, 10.44, 15.17, 18.00, 22.08, 26.76, 38.70, and 56.21 Ω (TRU components, Vishay Intertechnology). Each resistor is inserted as long as the voltage reaches the plateau and/or maximally for 2 min.

## Data Availability

All data needed to evaluate the conclusions in the paper are present in the paper and/or the Supporting Information. Additional data related to this paper are available on request.

## Supporting Information Available

Resistance calculations; electromotive force and voltage calculations; gross, pumping, and net power density calculations; fluidics; passive mixers design; ERS fluidics; performance of the RED device *n* = 5 with different channel heights *h*; *U*–*I* curve; engineering the RED device *n* = 5 with different channel heights *h*; pressure drop of HC and LC vs *V̇*, vs *h*; enhanced mixing at the membrane/saline solution interface; engineering ERS fluidics in RED device *n* = 5 with *h* = 300 μm; and engineering ERS composition in RED device *n* = 5 with *h* = 300 μm (PDF)

## Nomenclature

*A*: area of the membrane/saline solution interface (one-side), m^2^
A: constant for Hagen-Pouiseulle flow
*a*: width of the grooves, m
B: constant for the Fickian diffusion process normal to the flow direction
*b*: width of ridges, m
*c*: concentration of the cation or anion in the HC and LC, M
CP: cell pair
*d*: depth of the grooves, m
Δ*p*_LC/HC/ERS_: pressure drop across the device, respectively, for LC, HC, and ERS, bar
*d*_h_: characteristic length of a fluid channel with a rectangular cross section equals the hydraulic diameter, m
EMF: electromotive force, V
ERS: electrode rinse solution
*f*_obs_: obstruction factor
*F*: Faraday constant, C mol^–1^
*h*: membrane separation distance/channel height, μm
HC: high-concentration feed solution
*I*: current output, mA
*L*: channel length, m
LC: low-concentration feed solution
*n*: number of cell pairs, -
*N*: number of grooves per half-cycle, -
*P*: asymmetric factor, equals *w*_l_/*w*, -
*Pd*: power density, W CP^–1^ m^–2^
*Pd*_gross_: gross power density, W CP^–1^ m^–2^
*Pd*_in_: pumping power density, W CP^–1^ m^–2^
*Pd*_net_: net power density, W CP^–1^ m^–2^
*P*_wet_: cross-sectional perimeter wetted by the shear stress, m
*r*: resistance of one cell pair, Ω
*R*: universal gas constant, J mol^–1^ K^–1^
*R*_AEM/CEM_: resistance of the anion-/cation-exchange membrane, respectively, Ω
*Re*: Reynolds number (for LC, HC, and ERS), -
RED: reverse electrodialysis
*R*_elec_: resistance of the electrode compartment (consisting of the shielding membrane, the ERS, and the electrodes themselves), Ω
*R*_HC/LC_: resistance of the HC/LC compartment, respectively, Ω
*R*_i_: internal resistance of RED stack, Ω
*R*_non-ohmic_: non-Ohmic resistance of RED stack, Ω
*R*_ohmic_: Ohmic resistance of RED stack, Ω
*s*: scan rate, mV s^–1^
*T*: temperature, K
τ: residence time of ERS within the electrode compartment, s
*U*_max_: RED device voltage output, V
*U*_red-ox_: potential difference of the oxidation–reduction reaction of ERS, V
*V*: electrode volume, m^3^
*V̇*: volumetric flow rates for LC, HC, and ERS, respectively, m^3^ s^–1^
*w*: channel width, m
*w*_l_: width of the long arm, m
*w*_s_: width of the short arm, m
*z*: valence of the ion, -
α_CEM/AEM_: apparent permselectivity of AEM and CEM, respectively, -
γ: activity coefficient of the cation or anion in the HC and LC, -
η: fluid dynamic viscosity, Pa s
θ: groove intersection angle, °
ρ: fluid density, kg m^–3^
σ_HC/LC_: ionic conductivity of respectively, HC and LC compartments, respectively, S m^–1^
υ: fluid velocity, m s^–1^
