# Supplementary material for: Engineering Electrode Rinse Solution Fluidics for Carbon-Based Reverse Electrodialysis Devices
Source: ACS Appl Mater Interfaces. 2023 Oct 9;15(41):48826–37. doi: 10.1021/acsami.3c10680 (PMC10591279; doi:10.1021/acsami.3c10680)
Supplement: Supplementary file 1 — am3c10680_si_001.pdf [file am3c10680_si_001.pdf]

Supporting Information

Engineering Electrode Rinse Solution Fluidics for Carbon-based Reverse Electrodialysis Devices

Anetta Platek-Mielczarek[1], *Johanna Lang*[1], *Feline Töpperwien*[1], *Dario Walde*[1], *Muriel Scherer*[1], *David P. Taylor*[2], *Thomas M. Schutzius*[1,3,*]

[1]Laboratory for Multiphase Thermofluidics and Surface Nanoengineering, Department of Mechanical and Process Engineering, ETH Zurich, Sonneggstrasse 3, CH-8092 Zurich, Switzerland

[2]Laboratory of Thermodynamics in Emerging Technologies, Department of Mechanical and Process Engineering, ETH Zurich, Sonneggstrasse 3, Zurich, 8092, Switzerland

[3]Department of Mechanical Engineering, University of California, Berkeley, California 94720, USA

Thomas M. Schutzius ORCID: 0000-0003-3309-3568

Anetta Platek-Mielczarek ORCID: 0000-0001-6231-3908

David P. Taylor ORCID: 0000-0001-9759-4084

Muriel Scherer ORCID: 0000-0003-3777-8206

[*] To whom correspondence should be addressed: Prof. Thomas M. Schutzius, Department of Mechanical Engineering, University of California at Berkeley, Berkeley, California 94720, USA; E-mail: tschutzius@berkeley.edu

Supporting Information – Notes

Supplementary Note 1

Resistance calculations

The internal resistance of the RED stack (R_i) consists of several resistances, divided into ohmic resistances (R_{ohmic}) and non-ohmic resistances ($R_{non-ohmic}$):

$$R_i = R_{ohmic} + R_{non-ohmic} \cdot \qquad \text{(Eq. S1)}$$

Ohmic resistance refers to the resistances of all RED components: HC and LC saline solution compartments, AEM, CEM (all of which stands for cell pair resistance), AEM shielding membrane, ERS, electrodes and electrode connections (all of which stands for electrode compartment resistance). Therefore, the cell pair resistance (r) equals:

$$r = R_{AEM} + R_{CEM} + R_{HC} + R_{LC} \quad , \qquad \text{(Eq. S2)}$$

where membrane resistances (R_{AEM} and R_{CEM}) are material properties, and HC and LC compartments resistances (R_{HC} and R_{LC}, respectively) can be calculated using the formula:

$$R_{HC/LC} = f_{obs} \frac{h}{\sigma_{HC/LC} A} \quad , \qquad \text{(Eq. S3)}$$

where $\sigma_{HC/LC}$ (S m^{-1}) is the ionic conductivity of HC and LC compartments, respectively, h (m) is the channel height, A (m^2) is the area of direct one-side contact between membrane and HC and LC, and f_{obs} is an obstruction factor (for channels without spacer f_{obs} equals to 1).

For calculating the resistance of HC and LC compartment the average conductivity of the solution within the cell is considered. For this reason, the solutions' conductivity is measured *before* being pumped into the RED device and *after* exiting the RED stack, enabling average calculations. The obtained values for HC compartment are: before σ_{HC}= 87 mS cm^{-1} and after σ_{HC}= 44 mS cm^{-1} leading to an average value of 66 mS cm^{-1}. For LC compartment are: before σ_{LC}= 1 mS cm^{-1} and after σ_{LC}= 53 mS cm^{-1} leading to an average value of 27 mS cm^{-1}. Interface

area equals membrane area in direct contact with HC or LC, A, equals 110 cm^2. Given that, R_{HC} is calculated to be 4.2 mΩ and R_{LC}= 10.5 mΩ for n= 1 with h= 300 μm. Specific membrane resistance (Ω m^2) is given by the membrane producer, 0.3 mΩ m^2 and needs to be multiplied with the membrane area, A, to obtain the membrane resistance (Ω). Thus, $R_{AEM} = R_{CEM} = 27.3$ mΩ.

$$r = 27.3 + 27.3 + 4.2 + 10.5 = 69.3\ (\mathrm{m\Omega})\ .$$

The resistance of the whole electrode compartment can be calculated by extrapolating the resistance values from various numbers of cell pairs to a RED stack with n= 0. Experiments are conducted for n= 1 – 6.1 Ω, and n= 5 – 17.1 Ω, leading to a n= 0 value of 3.4 Ω for h= 300 μm channel height. Given that, R_{ohmic} can be calculated:

$$R_{\mathrm{ohmic}} = n\,r + R_{\mathrm{elec}} \approx 3.75\ (\Omega)\ . \quad \text{(Eq. S4)}$$

Deviations between calculated and measured values are probably caused by deviations from theoretical assumptions, i.e., constant ionic conductivity within a channel, uniform distribution of HC and LC solutions within all fluid channels, etc.

Calculated ohmic resistances of the RED stack composed of different gaskets (channel heights, h) are summarized in Table S1.

Table S1. Ohmic resistance of various RED device with n= 1 and n= 5 and various h

	***h*= 100 μm**		***h*= 125 μm**		***h*= 300 μm**	
	n= 1	*n*= 5	*n*= 1	*n*= 5	*n*= 1	*n*= 5
**R_{ohmic}, Ω**	8.6	10.9	8.5	10.0	8.5	14.1

Supplementary Note 2

Electromotive force and voltage calculations

The electromotive force created at the membrane/saline solution interface can be calculated using the following formula:

$$EMF_{CEM} = \alpha_{CEM}\frac{RT}{zF}\ln\left(\frac{\gamma_{HC}^{+}c_{HC}^{+}}{\gamma_{LC}^{+}c_{LC}^{+}}\right) \qquad , \qquad \text{(Eq. S5)}$$

$$EMF_{AEM} = \alpha_{AEM}\frac{RT}{zF}\ln\left(\frac{\gamma_{HC}^{-}c_{HC}^{-}}{\gamma_{LC}^{-}c_{LC}^{-}}\right) \qquad , \qquad \text{(Eq. S6)}$$

where $\alpha_{CEM, AEM}$ is the apparent permselectivity of the membrane (-), R-universal gas constant (J mol^{-1} K^{-1}), T-temperature (K), z-valence of ion (-), F-Faraday constant (C mol^{-1}), γ-activity coefficient of cation or anion in the HC and LC, c-concentration of cation or anion in the HC and LC. Using α_{CEM}=0.96 and α_{AEM}=0.93, given by producer, R= 3.814 J mol^{-1} K^{-1}, T= 298 K, z= 1, F= 96 485 C mol^{-1}, c_{HC}= 1 M, c_{LC}= 0.01 M and γ_{HC}=γ_{LC} we get: EMF_{CEM}= 0.114 V and EMF_{AEM}= 0.110 V.

The total *EMF* of the RED device is equal to: $U_{max}=(EMF_{CAM}+EMF_{AEM})n$, and gives $U_{max,\ n=1}$= 0.224 V or $U_{max,\ n=5}$= 1.120 V theoretical maximal voltage.

Supplementary Note 3

Gross, Pumping and Net Power Density calculations.

Power, *P,* can be predicted using Ohm's law, combining voltage, *U*, current, *I*, and resistance, *R*:

$$P = \frac{U^2}{R} = U\,I = R\,I^2\,. \qquad \text{(Eq. S7)}$$

The power *P* (W) needs to be later recalculated considering the number of cell pairs, *n*, and one-side direct area between membrane and saline solution, *A*, to obtain gross power density, $Pd_{gross,}$ in mW CP^{-1} m^{-2} to enable comparison with experimental data and other literature reported values. For the voltage, one can consider *EMF* multiplied by number of cell pairs, *n*, 0.224 V · 5= 1.12 V. Maximum power is obtained when external load matches R_i, thus, R_i is taken into calculations. Computation of current, *I*, is difficult as Faraday's law cannot be applied straightforwardly, as no deposition or dissolution processes are ongoing at the electrode material. The redox system should run effectively, without deposition/dissolution reactions and it varies depending on the parameters, such as redox species concentration, activity, electrode material, porosity, flow conditions and many others. Therefore, for the purpose of computing the real RED stack power metrics maximum voltage and current have been considered for calculations using a semiempirical approach, leading to the feasible maximum power output of this device with various channel heights. Taking it into account that the theoretical Pd_{gross} equals 420 mW CP^{-1} m^{-2} for *h*= 300 μm, 347 mW CP^{-1} m^{-2} for *h*= 125 μm, and 222 mW CP^{-1} m^{-2} for *h*= 100 μm. Thus, gross power density can be plotted against channel height.

Theoretical pumping power (P_{in}) predictions use straightforward computation, as pressure drop, Δp, and flow rate, *V*, need to be considered for each solution flowing through RED device, i.e., LC, HC and ERS.

$$P_{in} = \Delta p\ V = R\ V^2 = \frac{4\,\eta\,l}{w\,h^3}\frac{1}{\frac{1}{3} - \frac{64\,h}{\pi^5\,w}\tanh\left(\frac{\pi\,w}{2\,h}\right)}\,Q^2 \qquad \text{(Eq. S8)}$$

This equation (Eq. S8) takes into account hydrodynamic resistance of rectangular channel and recalculate it to cylindrical pipe cross sectional diameter [1], η– fluid dynamic viscosity (Pa s), *L*– channel length (m), *w*– channel width (m), *h*– channel height (m), *V*– flow rate ($m^3\ s^{-1}$). Maximum power input is calculated for a pump setting with V_{HC}= V_{LC}= 0.21 cm^3 s^{-1} flow rate, that is maximal for HC and LC solutions, and V_{ERS}= 0.05 $cm^3\ s^{-1}$. P_{in} divided by *n* and *A* gives Pd_{in}.

The net power density is obtained when pumping power density is subtracted from gross power density, $Pd_{net}= Pd_{gross} - Pd_{in}$. It directly shows the available power that can be used by external devices.

Supplementary Note 4

Fluidics

The Reynolds number (Re) of a fluid can be calculated using the equation:

$$Re = \frac{\rho\, v\, L}{\eta}\,, \qquad \text{(Eq. S9)}$$

where: ρ–fluid density (kg m^{-3}), *υ*–fluid velocity (m s^{-1}), *L*–characteristic length of the fluid channel (m), η–fluid dynamic viscosity (Pa s).

The characteristic length of a fluid channel with rectangular cross section equals the hydraulic diameter d_h (m), defined as:

$$d_h = \frac{4\,A}{P_{\text{wet}}} = \frac{4\,w\,h}{2\,w + 2h}\,, \qquad \text{(Eq. S10)}$$

where: *A*–cross-sectional area of the channel (m^2), P_{wet}–cross-sectional perimeter wetted by the shear stress (m), *w* and *h*– channel width and height (m). For calculations density and viscosity of HC and LC solutions were assumed to be the same as for pure water (298 K), namely: ρ= 999.8 kg m^{-3} and η= 1.79 mPa s. Width of channel *w*= 1.3 cm, height, *h*=100, 125 or 300 μm, respectively. Given that, the Reynolds number is calculated for each system with *n*= 5 and equals: 0.7 for *h*= 300 μm; 0.4 for *h*= 125 μm and 0.7 for *h*= 100 μm. Thus, the Re number for all experiments of RED with *n*= 5 independently on a *h* is lower than 1.

Supplementary Note 5

Passive mixers design

Passive micromixers increase Pd_{gross}, without substantial cost increase—as no external energy input to run them is required. Passive micromixers disturb the fluid flow by physical manner [2]. For this purpose, one or more walls of the fluid channel are designed with notches or protrusions, often in the form of grooves and ridges. One of these structure designs is the staggered herringbone mixer (SHM), which generates a chaotic flow even at low Reynolds numbers, Re < 100 [3]. We focus on this mixing structure as Re <1 for our RED device with n= 5. SHM structures consist of V-shaped grooves that are installed at regular intervals in the fluid channel. The V is asymmetrical and has a long and a short arm. The term SHM-cycle is used when several grooves are installed one after the other and the orientation of the asymmetrical grooves changes halfway through one cycle (two half-cycles). The geometrical parameters that fully define the SHM structures are:

- height of the fluid channel h (m)
- width of the fluid channel w (m)
- dept of the grooves d (m)
- width of the grooves a (m)
- width of the ridges b (m)
- groove intersection angle θ (°)
- width of the short arm w_s (m)
- width of the long arm w_l (m)
- the asymmetric factor $P = w_l/w$ (-)
- the number of grooves per half-cycle N (-)
- the fraction of the membrane area that is covered by the ridges z (-).

Different studies shows that the mixing effect of the SHM is strongly dependent on the ratio of channel height to channel width *h/w* and on the ratio of groove width to ridge width *b/a*. The specific model is proposed to calculate the optimum groove width to ridge with ratio *b/a* [3], while the channel height to width ratio *h/w* seems to be optimal in the range of 0.46-0.48 [4, 5]. The asymmetric factor *P* should be 2/3 as at this value most of the cross-sectional area is involved in the chaotic flow [6]. The influence of the groove intersection angle θ has been addressed in several papers, and suggested optimum value lies between 36.8◦ [5] and 51.4◦ [4]. A smaller angle leads to a greater pressure drop in the channel [5]. Most of the papers state that the number of grooves is found to only have a minor [4] or no effect at [7, 8] all onto the mixing performance. The fraction of the wall that is covered with the mixing promoter does not play a role in most of the applications (e.g., heat/mass transfer [9]) but when used in RED it has an influence on the performance of the system. If part of the membrane is covered, this impedes contact and ion exchange between the feed water and the membrane interface (so-called shadow effect) [10]. The shadow effect is more prominent the higher the concealed fraction [11], so *z* should be kept as low as possible.

Passive micromixers could decrease resistance of the feed solutions, especially LC . Thus, their could find their potential application in RED device.

Supplementary Note 6

ERS Fluidics

The scan rate s (mV s^{-1}) from fundamental ERS characterization can be converted into a residence time τ (s) of the ERS in the electrode compartment using the expected voltage:

$$\tau = \frac{EMF}{s} \quad , \quad \text{(Eq. S11)}$$

when using theoretical values, it gives us 112 s of residence time of ERS in the electrode compartment to operate in the similar conditions as 10 mV s^{-1}. Knowing the dimensions of the electrode compartment–fully filled with carbon-felt electrode–channel length L (m), channel width w (m), channel height h (m), we can calculate the flow rate $\dot{V}_{ERS}$ (m^3 s^{-1}):

$$\dot{V}_{ERS} = \frac{L\,w\,h}{\tau} \quad , \quad \text{(Eq. S12)}$$

this gives us a flow rate of 0.25 cm^3 s^{-1} that is higher than maximum peristaltic pump settings (0.21 cm^3 s^{-1}). Given this, flow rates used during experiments are: 0.21 cm^3 s^{-1} – resembling 10 mV s^{-1}, 0.14 cm^3 s^{-1} – resembling 5 mV s^{-1}, and 0.05 cm^3 s^{-1} –resembling 2 mV s^{-1}. The higher ERS flow rate, the higher Pd_{in} for the RED device, which needs to be considered to find a sweet spot based on Pd_{net}.

As the flow rate of ERS is controlled during the RED device operation, one can calculate the residence time of the ERS within one electrode compartment (knowing the volume of the electrode, h, w, L, and flow rate, $\dot{V}_{ERS}$). The residence time for the highest flow rate applied ($\dot{V}_{ERS}$= 0.21 cm^3 s^{-1}) is 134 s, for $\dot{V}_{ERS}$= 0.14 cm^3 s^{-1} – 256 s, and for $\dot{V}_{ERS}$= 0.05 cm^3 s^{-1} is 562 s. Diffusivity of the redox species – iron cations equals: D_{Fe2+}= 7.19·10^{-6} cm^2 s^{-1}, and D_{Fe3+}= 6.04·10^{-6} cm^2 s^{-1} what gives an average diffusivity value for Fe^{2+}/Fe^{3+} $\overline{D}$= 6.62·10^{-6} cm^2 s^{-1}. We can calculate the diffusion time that is needed for redox species to penetrate into carbon porosity and for average diffusion coefficient and electrode dimensions it is ca. 49000

s, more than 13 h. Based on this, we propose diffusion limited ERS operation at carbon electrodes highlighting engineering of this compartment as a crucial thing in order to increase the power density and also showing that very low pumping conditions should be beneficial for Pd_{gross} increase (potential with longer time for redox species to diffuse towards electrode bulk) and lower Pd_{in} needed to pump the ERS at flow rates $V_{ERS}< 0.05$ cm^3 s^{-1}.

Supplementary Information – Figures

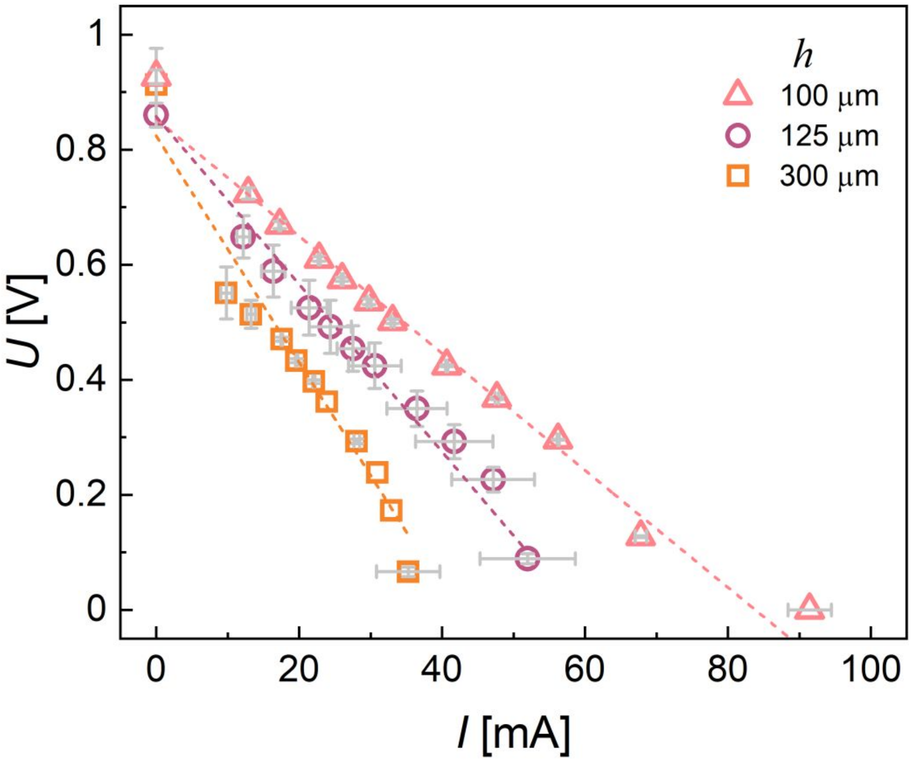

Figure S1. Performance of the RED device n= 5 with different channel height, h. LC= 0.01 M NaCl, and HC= 1 M NaCl both with $V_{LC, HC}$= 0.21 cm^3 s^{-1}, ERS= 20 mM Fe^{2+}=Fe^{3+}, 0.01 M NaCl with V_{ERS}= 0.05 cm^3 s^{-1}, represented in U-I curve: voltage, U, vs. current, I.

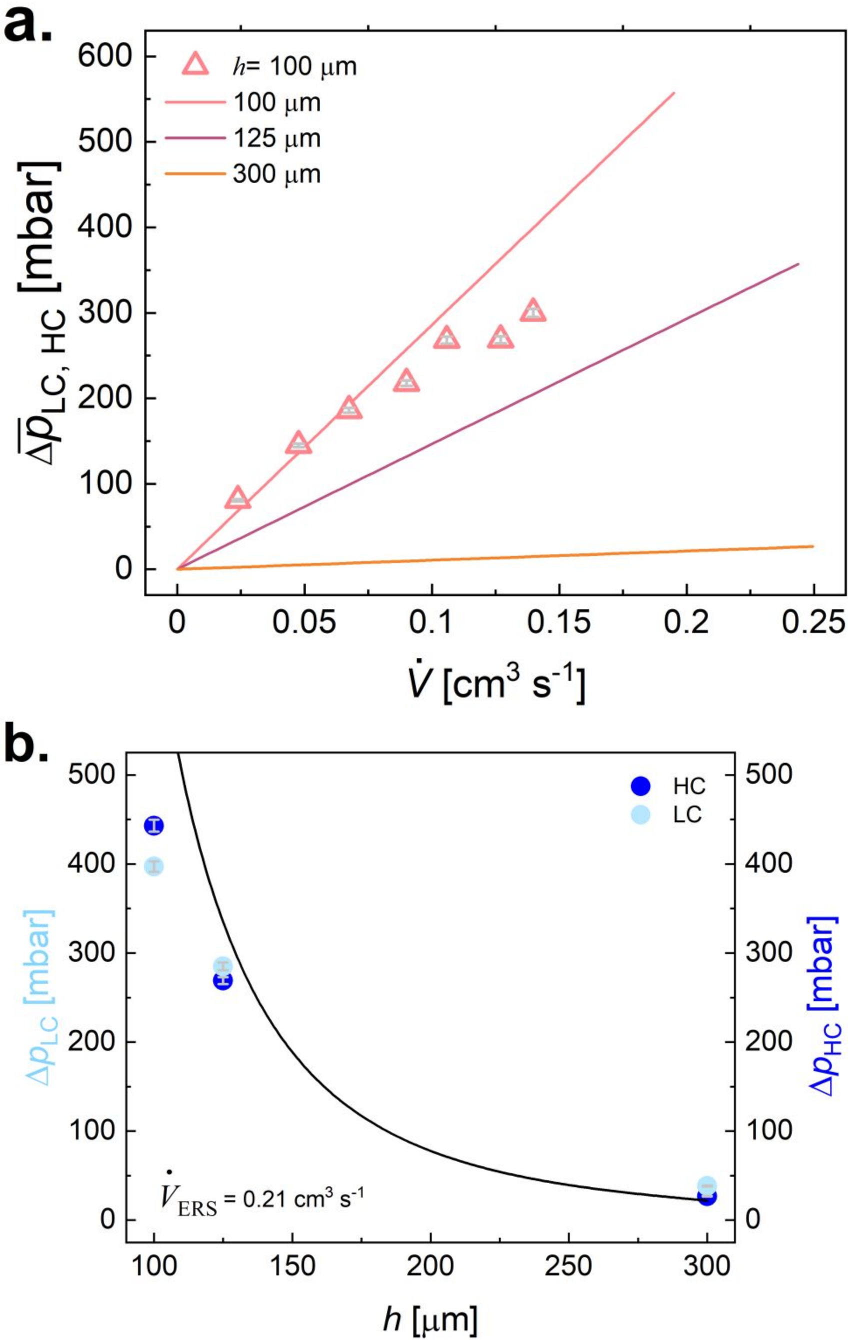

Figure S2. **Engineering RED device *n*= 5 with different channel height, *h*: a)** Pressure drop computation for average pressure drop in LC and HC compartment (calculated from equation $\Delta p = (12\eta L V_{LC,\ HC})/h^3$, with dynamic viscosity, η= 1.79 mPa s, channel length L= 0.865 cm, and channel height, *h*) for various flow rates, $V_{LC,\ HC}$, and membrane separation distance, *h*, together with experimental data obtained with *h*= 100 μm for average of LC and HC solutions ($\Delta p_{LC,\ HC} = (\Delta p_{LC} + \Delta p_{HC})/2$); **b)** pressure drop of LC and HC for various channel height, *h*.

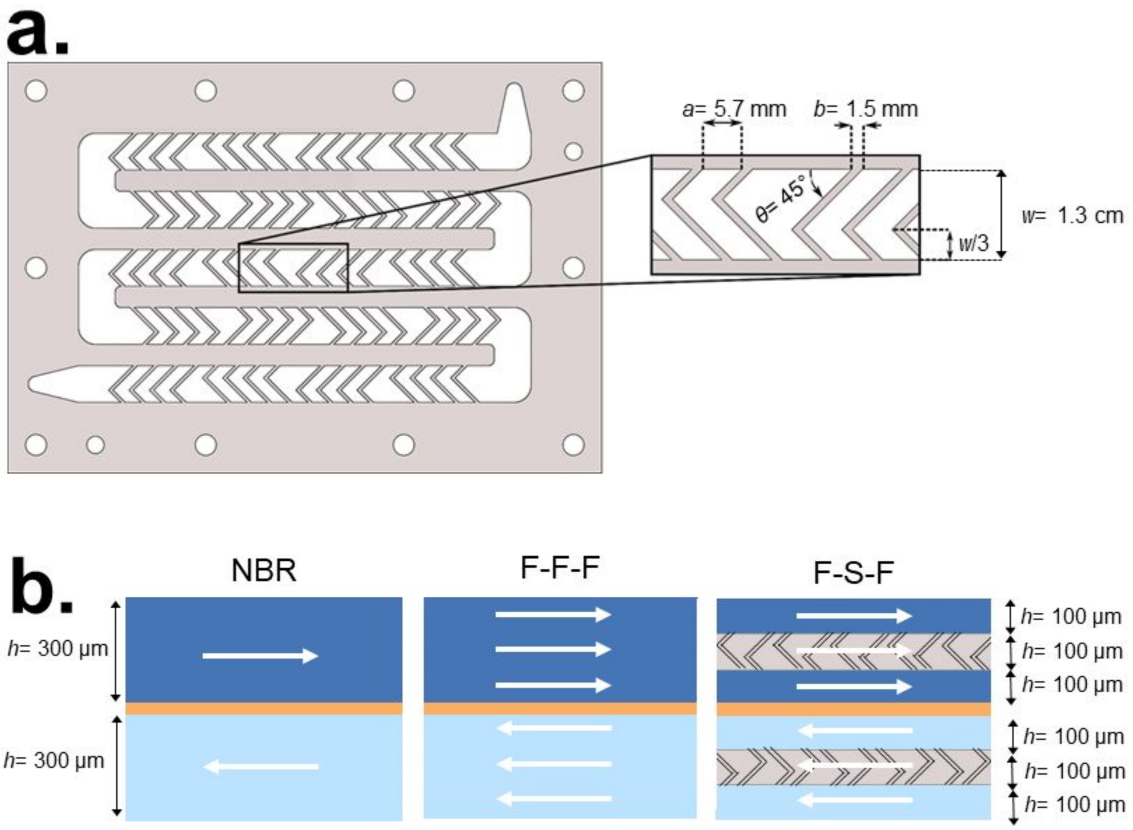
a.
a= 5.7 mm
b= 1.5 mm
θ= 45°
w= 1.3 cm
w/3
b.
NBR
F-F-F
F-S-F
h= 300 μm
h= 300 μm
h= 100 μm
h= 100 μm
h= 100 μm
h= 100 μm
h= 100 μm
h= 100 μm

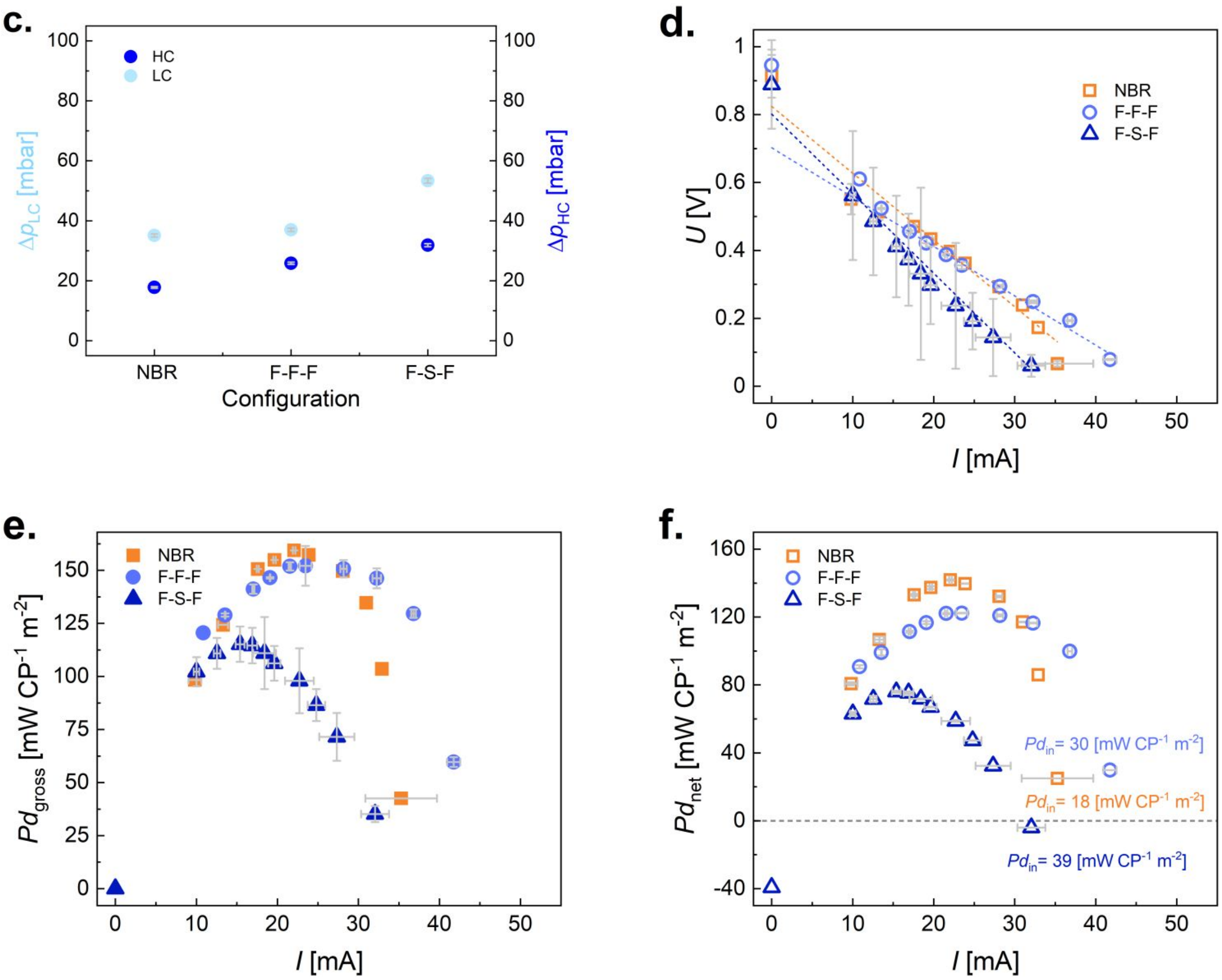

Figure S3. **Enhanced mixing at the membrane/saline solution interface.** Design of passive mixer structure for fixed membrane separation distance (*h*= 300 μm) and various saline solution channel configurations in a RED device with *n*=5: **a)** passive mixer design with herringbone structure designed for a channel width, *w*= 1.3 cm; **b)** schematic of the studied configurations within LC and HC saline solution compartment with maintained *h*= 300 μm (orange line represents AEM and/or CEM with a thickness of 75 μm) by applying one NBR gasket (300 μm) - NBR, three Folex gaskets (3x 100 μm) – F-F-F and Folex passive mixer sandwiched between two Folex gaskets (3x 100 μm) – F-S-F; **c)** pressure drop recorded for HC and LC compartments for three configurations studied (NBR, F-F-F and F-S-F); **d)** *U vs. I*; **e)** Pd_{gross} *vs*. I; **f)** Pd_{net} *vs*. *I* with Pd_{in} values.

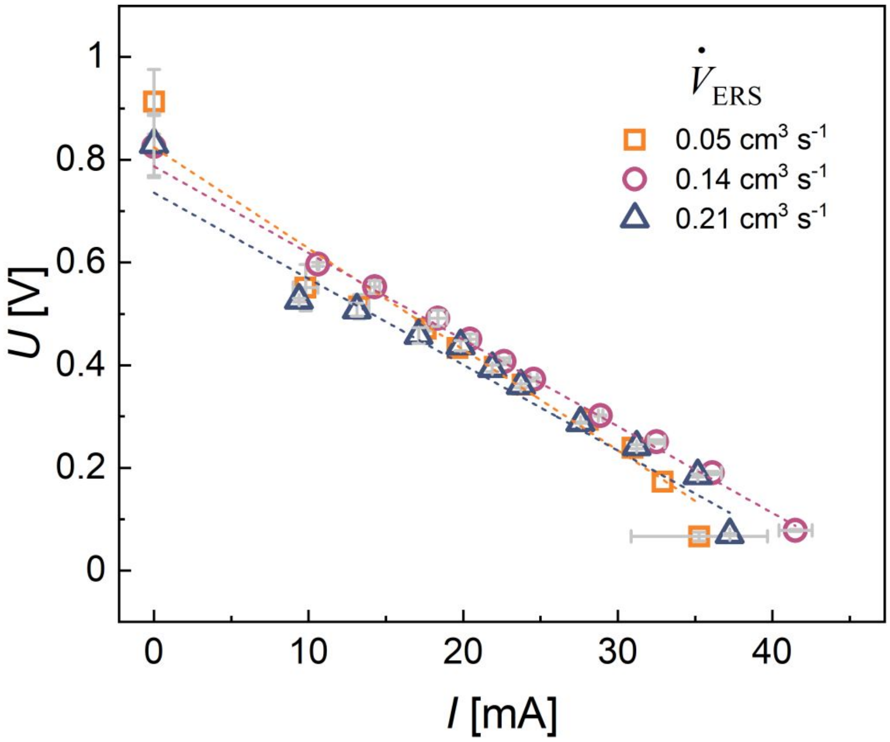

Figure S4. **Engineering of the ERS fluidics in RED device *n*= 5 with *h*=300 µm.** LC= 0.01 M NaCl, and HC= 1 M NaCl both with $V_{LC,\ HC}$= 0.21 cm^3 s^{-1}, ERS= 20 mM Fe^{2+}=Fe^{3+}, 0.01 M NaCl with V_{ERS}= 0.05 cm^3 s^{-1}, 0.14 cm^3 s^{-1} and 0.21 cm^3 s^{-1}, represented in *U-I* curve.

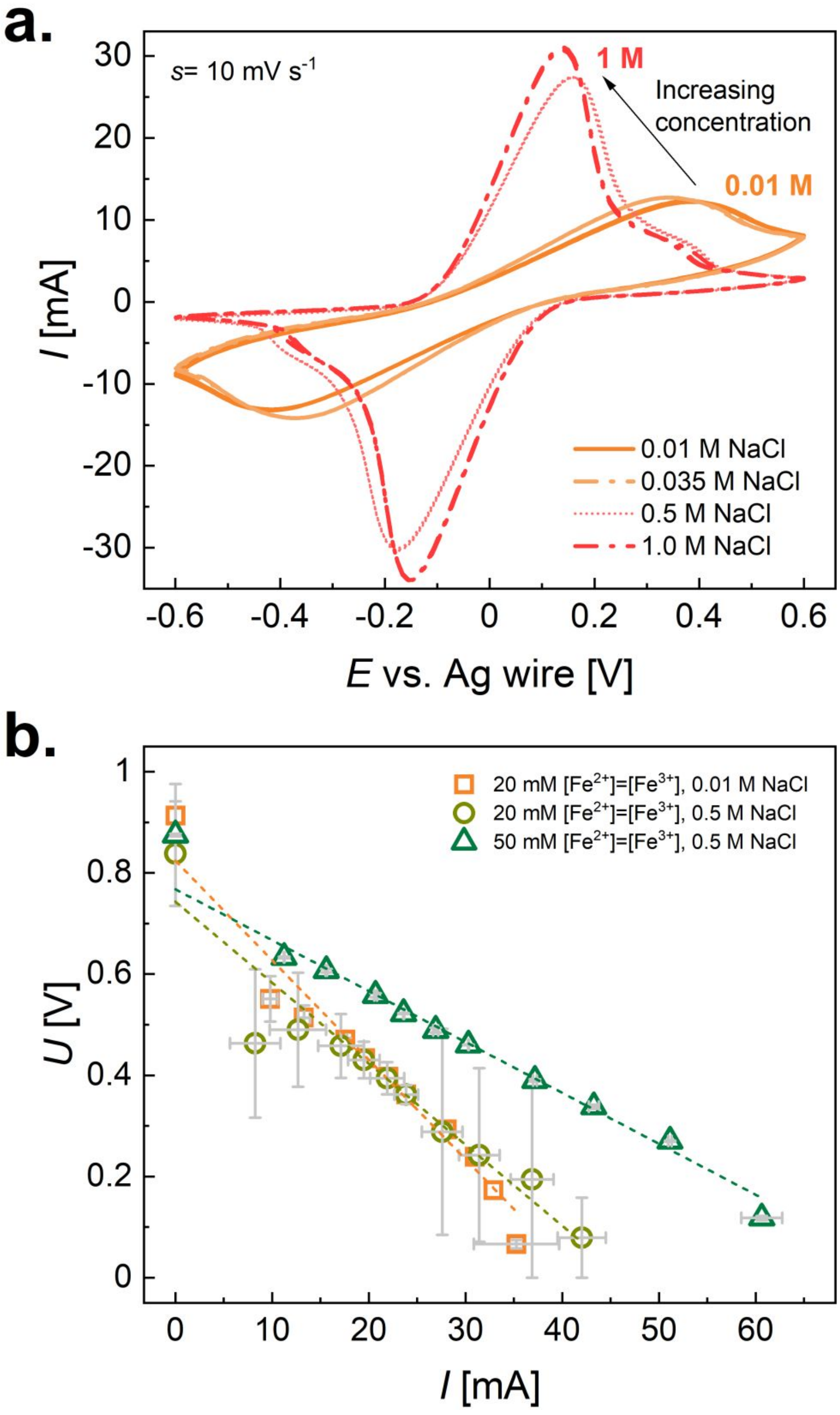
a.
s= 10 mV s-1
1 M
Increasing concentration
0.01 M
I [mA]
E vs. Ag wire [V]
0.01 M NaCl
0.035 M NaCl
0.5 M NaCl
1.0 M NaCl
b.
20 mM [Fe2+]=[Fe3+], 0.01 M NaCl
20 mM [Fe2+]=[Fe3+], 0.5 M NaCl
50 mM [Fe2+]=[Fe3+], 0.5 M NaCl
U [V]
I [mA]

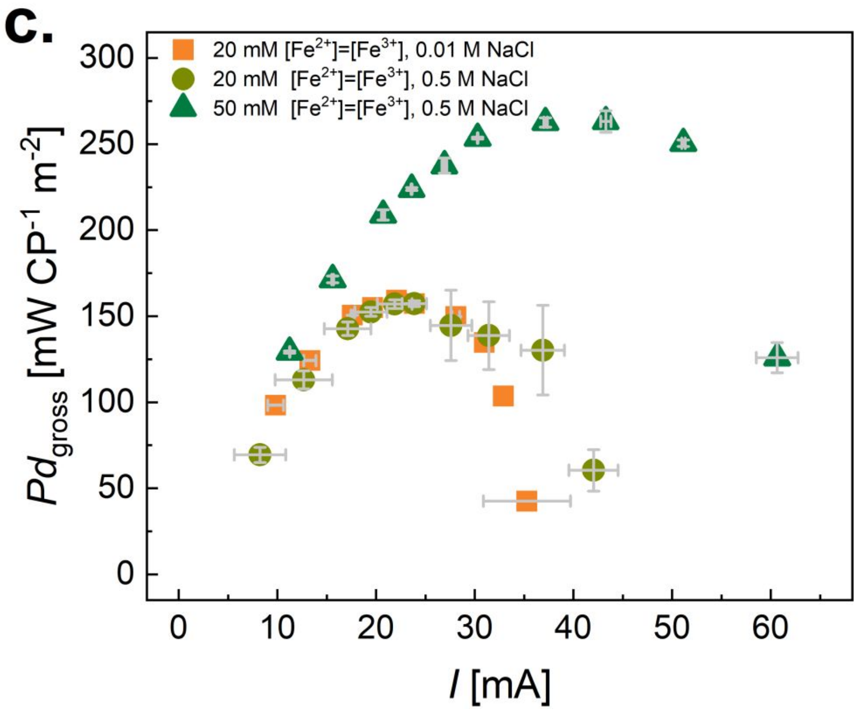

Figure S5. **Engineering of the ERS composition in the RED device *n*= 5 with *h*=300 µm**. LC= 0.01 M NaCl, and HC= 1 M NaCl both with $V_{LC, HC}$= 0.21 cm^3 s^{-1}, ERS with V_{ERS}= 0.05 cm^3 s^{-1} and various composition represented in: **a)** *U-I* curve; **b)** Pd_{gross} *vs*. *I*.

Supplementary Information References

[1] Taylor, D.P.; Kaigala, G.V. Fluidic Bypass Structures for Improving the Robustness of Liquid Scanning Probes. IEEE Trans. Biomed. Eng. 2019, 66 (9), 2491-2498. DOI: 10.1109/TBME.2018.2890602
[2] Bayareh, M.; Ashani, M.N.; Usefian, A. Active and passive micromixers: A comprehensive review. Chem. Eng. Process. 2020, 147, 107771. DOI: 10.1016/j.cep.2019.107771
[3] Lynn, N.S.; Dandy, D.S. Geometrical optimization of helical flow in grooved micromixers. Lab Chip 2007, 7 (5), 580-587. DOI: 10.1039/B700811B
[4] Hossain, S.; Husain, A.; Kim, K.-Y. Shape optimization of a micromixer with staggered-herringbone grooves patterned on opposite walls. Chem. Eng. J. 2010, 162 (2), 730-737. DOI: 10.1016/j.cej.2010.05.056
[5] Ansari, M.A.; Kim, K.-Y. Shape optimization of a micromixer with staggered herringbone grooves. Chem. Eng. Sci. 2007, 62 (23), 6687-6695. DOI: 10.1016/j.ces.2007.07.059
[6] Stroock, A.D.; Stephan, K.W.D.; Ajdari, A.; Mezić, I.; Stone, H.A.; Whitesides, G.M. Chaotic Mixer for Microchannels. Science 2002, 295 (5555), 647-651. DOI: 10.1126/science.1066238
[7] Li, C.; Chen, T. Simulation and optimization of chaotic micromixer using lattice Boltzmann method. Sens. Actuators B Chem. 2005, 106 (2), 871-877. DOI: 10.1016/j.snb.2004.09.006
[8] Cortes-Quiroz, C.A.; Azarbadegan, A.; Zangeneh, M.; Goto, A.; Analysis and multi-criteria design optimization of geometric characteristics of grooved micromixer. Chem. Eng. J. 2010, 160 (3), 852-864. DOI: 10.1016/j.cej.2010.02.029
[9] Cho, H.H.; Wu, S.J.; Kwon, H.J. Local Heat/Mass Transfer Measurements in a Rectangular Duct With Discrete Ribs. J. Turbomach. 2000, 122 (3), 579-586. DOI: 10.1115/1.1303049
[10] Zhang, K.; Wang, M.; Gao, C. Ion conductive spacers for the energy-saving production of the tartaric acid in bipolar membrane electrodialysis. J. Membr. Sci. 2012, 387 (1), 48-53. DOI: 10.1016/j.memsci.2011.10.012
[11] Kim, H.-K.; Lee, M.-S.; Lee, S.-Y.; Choi, Y.-W.; Jeong, N.-J.; Kim, C.-S. High power density of reverse electrodialysis with pore-filling ion exchange membranes and a high-open-area spacer. J. Mat. Chem. A 2015, 3 (31), 16302-16306. DOI: 10.1039/C5TA03571F
